# Cancer and hepatic steatosis

**DOI:** 10.1016/j.esmoop.2021.100185

**Published:** 2021-06-15

**Authors:** R. Paternostro, W. Sieghart, M. Trauner, M. Pinter

**Affiliations:** 1 Division of Gastroenterology and Hepatology, Department of Internal Medicine III, Medical University of Vienna, Vienna, Austria; 2 Liver Cancer (HCC) Study Group Vienna, Medical University of Vienna, Vienna, Austria

**Keywords:** non-alcoholic fatty liver disease, hepatocellular carcinoma, cancer, hepatic steatosis, chemotherapy-induced steatohepatitis

## Abstract

Non-alcoholic fatty liver disease (NAFLD) is a highly prevalent and increasing liver disease, which encompasses a variety of liver diseases of different severity. NAFLD can lead to liver cirrhosis with all its complications as well as hepatocellular carcinoma (HCC). Steatosis of the liver is not only related to obesity and other metabolic risk factors, but can also be caused by several drugs, including certain cytotoxic chemotherapeutic agents. In patients undergoing liver surgery, hepatic steatosis is associated with an increased risk of post-operative morbidity and mortality. This review paper summarizes implications of hepatic steatosis on the management of patients with cancer. Specifically, we discuss the epidemiological trends, pathophysiological mechanisms, and management of NAFLD, and its role as a leading cause of liver cancer. We elaborate on factors promoting immunosuppression in patients with NAFLD-related HCC and how this may affect the efficacy of immunotherapy. We also summarize the mechanisms and clinical course of chemotherapy-induced acute steatohepatitis (CASH) and its implications on cancer treatment, especially in patients undergoing liver resection.

## Highlights

• Non-alcoholic fatty liver disease can lead to cirrhosis with all its complications, including hepatocellular carcinoma.
• Chemotherapy-associated acute steatohepatitis is a side-effect of chemotherapeutic agents and may limit treatment options.
• In this review we summarize current clinical concepts of NAFLD and CASH that help clinicians in their clinical practice.

## Introduction

Obesity—a major health problem globally^1^—is not only associated with the development of cardiovascular complications,^2^ but also increases the risk for liver diseases, including non-alcoholic fatty liver disease (NAFLD) and liver cancer.^3^^,^^4^ Obesity is also associated with at least 12 other tumor types (i.e. esophageal, gastric, colorectal, gallbladder, pancreatic, breast, corpus uteri, ovarian, renal cell, thyroid, multiple myeloma, and meningioma).^5^ NAFLD encompasses a variety of liver diseases of different severity, ranging from simple steatosis to non-alcoholic steatohepatitis (NASH), and eventually results in liver cirrhosis and/or hepatocellular carcinoma (HCC) in a significant proportion of affected individuals.^6^ Underlying liver cirrhosis impacts on the prognosis and management of patients with HCC, as more advanced liver function impairment limits therapeutic options and worsens outcome.^7^

Hepatic steatosis is not only related to obesity and other metabolic risk factors, but can also occur as a feature of drug-induced liver injury.^8^^,^^9^ Several drugs are known to promote hepatic fat accumulation, including certain cytotoxic chemotherapeutic agents.^10^ This can have implications for the treatment of cancer patients, especially for those undergoing liver surgery for metastatic disease.^11^

In this review, we discuss the epidemiology, pathophysiology, and management of NAFLD, and its role as a leading cause of liver cancer. We summarize emerging evidence indicating that NAFLD may be associated with reduced efficacy of immunotherapy in HCC. Moreover, we elaborate on the mechanisms and clinical course of chemotherapy-induced steatohepatitis and its implications for the management of cancer patients.

## NAFLD: epidemiology, pathophysiology, diagnosis, and treatment

NAFLD has by some been referred to as the most rapidly emerging liver disease of the 21st century, with prevalence rates ranging between 23% and 32% in most parts of the world.^3^^,^^12^^,^^13^ Based on histology, NAFLD can range from simple steatosis without evidence of hepatocellular injury—referred to as non-alcoholic fatty liver (NAFL)—to steatosis with hepatic inflammation and hepatocyte injury, reflected by ballooning—referred to as non-alcoholic steatohepatitis (NASH).^14^ Risk factors for the development of NAFLD mainly include components of metabolic syndrome, such as diabetes mellitus, obesity, and hyperlipidemia.^14^ In consideration of the large contribution of metabolic risk factors to the evolvement of NAFLD and its disease severity, an international expert panel has recently proposed the new term ‘Metabolic Dysfunction-Associated Fatty Liver Disease’ (MAFLD) for patients with hepatic steatosis and type 2 diabetes or presence of at least two metabolic risk abnormalities.^15^^,^^16^ However, it remains unknown how quickly this newly proposed definition will be adapted into daily clinical practice. Genetic risk factors have also been associated with an increased risk for developing NAFL, NASH, and its associated complications such as advanced fibrosis, cirrhosis, and HCC, with a genetic variation in the patatin-like phospholipase domain-containing protein 3 (PNPLA3) being the most widely studied.^17^, ^18^, ^19^

In a patient with suspected NAFLD, thorough review of the medical history and exclusion of other obvious causes of chronic liver disease (i.e. the most common being viral hepatitis, alcoholic liver disease, autoimmune-associated liver disease, cholestatic liver disease, drug-induced liver disease) is mandatory.

Apart from distinguishing whether a patient has NAFL or NASH, evaluation of the fibrosis stage is crucial, as fibrosis per se rather than the presence of NASH on liver biopsy seems to be the leading driver of major hepatic outcomes (i.e. liver-related mortality or hepatic decompensation such as ascites, hepatic encephalopathy, or variceal bleeding).^20^^,^^21^ Even though several non-invasive methods to evaluate fibrosis have proven to effectively rule-in/-out advanced fibrosis or cirrhosis in patients with NAFLD, liver biopsy remains the gold standard.^14^^,^^22^ Nevertheless, non-invasive methods such as vibration controlled transient elastography (VCTE; FibroScan, Echosense, Paris, France)^23^^,^^24^ and magnetic resonance elastography (MRE)^25^^,^^26^ or laboratory based scores such as FIB-4 or the NAFLD Fibrosis Score (NFS) are increasingly accepted and implemented in clinical routine.^14^^,^^22^^,^^27^

Given that the clinical benefit may be limited due to lacking therapeutic options outside of clinical trials, the indication of liver biopsy needs to be evaluated by a hepatology specialist, and pros and cons should be discussed with the patient. We usually perform non-invasive tests (VCTE or MRE, FIB-4 or NFS; see Table 1) first. If these are suggestive of advanced chronic liver disease (i.e. fibrosis stage ≥3), we recommend confirmatory liver biopsy, especially when evaluating eligibility for clinical trials.

**Table 1:** Bullet points on risk factors, diagnostic tools, histological readouts, and treatment options for patients with non-alcoholic fatty liver disease (NAFLD)

Prevalence?	NAFLD prevalence is estimated to range between 23% and 32% in most regions of the world. NAFLD summarizes two distinct disease courses: • NAFL = non-alcoholic fatty liver → steatosis, no inflammation • NASH = non-alcoholic steatohepatitis → steatosis and inflammation
Risk factors?	Lifestyle factors, obesity, diabetes mellitus, hyperlipidemia, arterial hypertension (= metabolic syndrome) and genetic risk factors (polymorphisms in PNPLA3, TM6SF2, HSD17B13)
Liver histology?	Should be evaluated for degree of steatosis (grade 0-3), inflammation (grade 0-3), ballooning (grade 0-2), and stage of fibrosis (stage 0-4) • Non-alcoholic fatty liver disease activity score (NAS) → NAS ≥5 points = highly suggestive of NASH • Advanced fibrosis = fibrosis stage ≥3 • Cirrhosis = fibrosis stage 4
Non-invasive tools?	• Abdominal ultrasound—steatosis? Liver surface indicative of cirrhosis (= irregular)? Suspicious liver nodules? • Vibration controlled transient elastography (i.e. FibroScan™) → Non-invasive staging of fibrosis • Magnetic resonance elastography → non-invasive staging of fibrosis • Laboratory based scores for ruling-in/-out advanced fibrosis: → FIB-4: formula containing the following variables: age (years), aspartate aminotransferase concentration (IU/l), alanine aminotransferase concentration (IU/l) and platelet count (∗10^9^/l); a score >3.25 is suggestive of advanced fibrosis → NAFLD Fibrosis Score: formula containing the following variables: age (years), body mass index (kg/m^2^), presence of impaired fasting glycaemia or diabetes (yes/no), aspartate aminotransferase concentration (IU/l), alanine aminotransferase concentration (IU/l), platelet count (∗10^9^/l), albumin concentration (g/dl); a score >0.675 is suggestive of advanced fibrosis
Staging/grading?	Stage and grade according to NAS and fibrosis stage, i.e. NASH patient with advanced fibrosis would be staged/graded: NAS 6, F3
Treatment options?	• Lifestyle factors, i.e. weight loss, Mediterranean diet, exercise • Treatment of comorbidities, i.e. metabolic syndrome (focus: glycemic control!) • Vitamin E or pioglitazone for selected patients only • Several phase III trials ongoing—referral to tertiary care center recommended for patients interested in participating in trials • If advanced fibrosis/cirrhosis → screen and treat associated complications (gastroesophageal varices, ascites, hepatic encephalopathy) • HCC surveillance in all cirrhotic NAFLD patients • HCC surveillance in selected non-cirrhotic NAFLD patients with high non-invasive fibrosis scores (VCTE, MRE, FIB-4, NAFLD Fibrosis Score)

FIB-4, fibrosis 4; HCC, hepatocellular carcinoma; HSD17B13, 17B-hydroxysteroid dehydrogenase type 13; MRE, magnetic resonance elastography; NAFLD, non-alcoholic fatty liver disease; NAS, non-alcoholic fatty liver disease activity score; PNPLA3, patatin-like phospholipase domain-containing protein 3; TM6SF2, transmembrane 6 superfamily 2 human gene; VCTE, vibration controlled transient elastography.

The liver specimen is graded according to the NAFLD activity score (NAS),^28^^,^^29^ which comprises the sum of the grade of steatosis (0-3), hepatocyte ballooning (0-2), and inflammation (0-3). Ranging from 0 to 8 points, a NAS score of ≥5 is highly suggestive of NASH.^29^ Liver fibrosis should be staged on a five-point scale: no fibrosis (stage 0), pericellular fibrosis (stage 1), pericellular and portal fibrosis (stage 2), bridging fibrosis (stage 3), or cirrhosis (stage 4),^28^ with ‘advanced fibrosis’ implicating stages 3 and 4.

Once diagnosis of NAFLD is made, treatment options aiming to reduce histopathological features as well as fibrosis should be discussed with the patient. Lifestyle interventions including weight loss and hypocaloric diet are the basis of all therapeutic interventions. Current guidelines recommend losing at least 3%-5% of body weight, as this was associated with improvement of steatosis. However, a reduction of around 7%-10% is usually needed to really impact histopathological features of NASH and fibrosis.^14^ In overweight/obese patients with advanced chronic liver disease, 16 weeks of diet and moderate exercise even reduced portal pressure.^30^ If this translates into a reduced number of hepatic events (i.e. liver-related mortality and/or hepatic decompensation) needs further evaluation. Besides weight loss, treatment of components of the metabolic syndrome, including hypertension, diabetes, and hyperlipidemia, represents another mainstay in the management of patients with NAFLD.^14^^,^^22^ Finally, several pharmacological treatments have been studied in patients with NASH, but only pioglitazone and vitamin E are recommended for selected patients by current practice guidelines.^14^^,^^22^

In patients with advanced fibrosis or cirrhosis, the presence of clinically significant portal hypertension (CSPH) needs to be evaluated. Hepatic venous pressure gradient (HVPG) measurement via hepatic vein catheterization represents the gold standard, and an HVPG of 10 mmHg or higher denotes CSPH. Indirect markers of CSPH include gastroesophageal varices in endoscopy as well as thrombocytopenia plus splenomegaly. Management includes evaluation and treatment of complications of CSPH (i.e. gastroesophageal varices, ascites, and hepatic encephalopathy).^31^, ^32^, ^33^

## HCC in NAFLD: incidence, screening, and treatment

Patients with advanced liver disease due to NAFLD show two main liver-related complications in the course of their disease—both leading to increased morbidity and mortality: (i) hepatic decompensation, including development of gastroesophageal varices and associated variceal bleeding, ascites, and hepatic encephalopathy, all of which almost exclusively occur in patients with advanced chronic liver disease (i.e. cirrhosis), and (ii) HCC, which may also occur in NAFLD patients without cirrhosis.^32^^,^^34^

Cancer-related mortality is among the top three causes of death in NAFLD patients.^14^^,^^35^ Overall cancer incidence is 783 per 100 000 person years in patients with NAFLD compared with 593 without NAFLD.^36^ However, this increased cancer incidence in NAFLD patients seems to be primarily driven by HCC development rather than extrahepatic cancers, as Simon and colleagues have shown that the contribution of extrahepatic cancers to the cancer incidence in NAFLD patients was modest at best.^37^ Liver cancer is the sixth most commonly diagnosed cancer worldwide and the fourth in leading causes for cancer-related death.^38^ HCC (75%-85%) and intrahepatic cholangiocarcinoma (10%-15%) include the majority of cases.^38^ Incidence and mortality rates are two to three times higher among men.^38^

Incidence rates of HCC in NAFLD-associated cirrhosis range between 1% and 3% per year,^39^, ^40^, ^41^ and on average, an incidence rate of >1.5% per year can be expected.^42^

HCC can also occur in non-cirrhotic NAFLD, although numbers are lower.^43^ HCC in non-cirrhotic livers is more frequent in those with metabolic syndrome and NAFLD compared with other etiologies.^43^^,^^44^ In a USA cohort of non-cirrhotic NAFLD patients, the HCC incidence rate was 0.21/1000 person years (= 0.02% annual risk).^45^ On the other hand, in patients with NAFLD-related HCC, up to 42%-54% developed in a non-cirrhotic liver, compared with only 2.8% in subjects with hepatitis C virus-associated HCC,^46^, ^47^, ^48^, ^49^ although a referral bias cannot be excluded.^6^

Apart from progression to advanced fibrosis/cirrhosis with the accompanying risk for HCC, diabetes mellitus—often associated with NAFLD—puts NAFLD patients at significant risk for developing HCC.^6^^,^^43^^,^^50^ Notably, antidiabetic drugs could have an impact on HCC risk. While metformin use is associated with a reduced risk of developing HCC, insulin may increase liver cancer risk.^51^

Genetic risk factors, mainly PNPLA3, transmembrane 6 superfamily 2 human gene (TM6SF2), and 17B-hydroxysteroid dehydrogenase type 13 (HSD17B13), have been associated with an increased HCC risk not only in NAFLD patients,^52^, ^53^, ^54^, ^55^ but also in the general population.^56^

Carcinogenesis in NAFLD is a complex, multifactorial process involving genetic and lifestyle factors (i.e. obesity, high fat diet) as well as small intestinal bacterial overgrowth. These factors induce cell death, cause genetic and epigenetic alterations, and activate pathways related to inflammation, cell proliferation, and hepatic energy metabolism. This results in the development of NASH and hepatic fibrosis, and eventually promotes hepatocarcinogenesis.^57^, ^58^, ^59^ Recent evidence suggests that obesity can promote HCC independently of NASH via STAT-3 signaling.^60^^,^^61^

**Figure 1:**
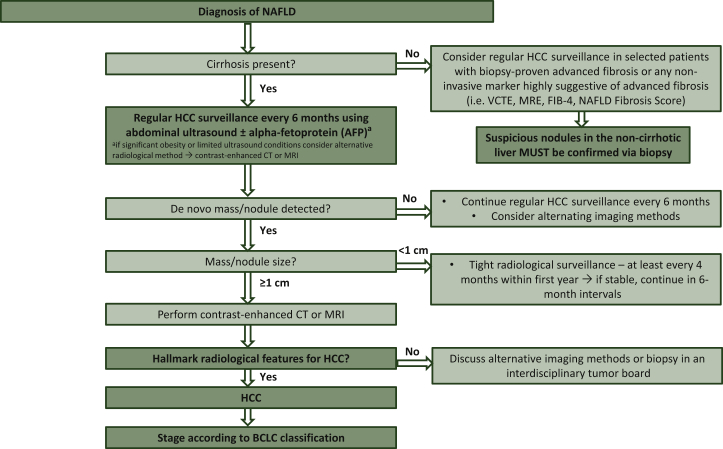
Surveillance algorithm for hepatocellular carcinoma in patients with non-alcoholic fatty liver disease. BCLC, Barcelona Clinic Liver Cancer; CT, computed tomography scan; FIB-4, Fibrosis-4; HCC, hepatocellular carcinoma; MRE, magnetic resonance elastography; MRI, magnetic resonance imaging; NAFLD, non-alcoholic fatty liver disease; VCTE, vibration controlled transient elastography.

Surveillance for HCC in NAFLD patients is recommended for individuals with liver cirrhosis and may be considered in non-cirrhotic patients with advanced fibrosis (fibrosis grade F3) based on an individual risk assessment (Figure 1).^34^^,^^42^ Screening should be carried out in 6-month intervals by ultrasound.^34^ While European guidelines do not recommend additional assessment of serum alpha-fetoprotein (AFP) during screening due to reasons of cost-effectiveness,^34^ its use is optional according to American guidelines.^62^ Other potential biomarkers, such as des-gamma-carboxy prothrombin (DCP), the AFP isoform AFP-L3, or glypican-3, have not been recommended for routine clinical use by current guidelines.^34^^,^^62^ Similarly, scores to detect early HCC (e.g. GALAD model^63^) need prospective validation before adoption in clinical routine.^62^

Notably, ultrasound depends on the operator and patient’s body composition, which can impair diagnostic accuracy, especially in overweight and obesity, a common clinical problem in NAFLD patients.^64^^,^^65^ Therefore, in cases where ultrasound is unreliable, practice guidelines recommend alternative imaging methods such as computed tomography (CT) scan or magnetic resonance imaging (MRI).^42^

If a nodule is detected on ultrasound, further steps depend on the size of the lesion. A nodule <1 cm in diameter should be followed at 4-month intervals in the first year, and if there is no increase in size or number, surveillance can be returned to the usual 6-month interval. For tumors <1 cm with typical HCC characteristics on CT or MRI, the optimal management has not been clarified yet. Thus, current guidelines recommend discussion within a local multidisciplinary tumor board. Tumors ≥1 cm need to be evaluated by multiphasic contrast-enhanced CT or MRI. In patients with liver cirrhosis, HCC can be diagnosed by imaging only if certain hallmarks are met.^34^ However, especially in small tumors, intrahepatic cholangiocarcinoma and HCC may show similar enhancement patterns,^66^ which could lead to a false diagnosis by imaging only. The lack of tissue samples also complicates the identification of predictive biomarkers to guide treatment decisions in HCC. Thus, at least in clinical studies, tumor biopsies should become mandatory.^67^ In non-cirrhotic livers, diagnosis must always be confirmed by histology.^34^

Staging and treatment of HCC depend on tumor burden, liver function, and performance status of the patient. The Barcelona Clinic Liver Cancer (BCLC) Staging System has been endorsed by current practice guidelines and recommends ablation, resection, and liver transplantation with curative intent for early stages, while palliative treatments (i.e. transarterial chemoembolization, systemic therapy) are indicated for intermediate-advanced stage HCC.^34^^,^^68^ Generally, surgical resection is recommended as treatment of choice in non-cirrhotic livers, which explains why NASH-associated HCC represents an emerging indication for resection.^34^ However, as up to 50% of NASH-HCCs occur in patients without cirrhosis, HCC is often diagnosed incidentally outside of screening programs and thus, at more advanced cancer stages with limited curative treatment options.^47^^,^^69^ Notably, NAFLD impairs functional recovery after liver resection, and the risk of major post-operative complications is higher in NAFLD patients—even if severe fibrosis is absent—compared with those with normal underlying liver.^70^, ^71^, ^72^ Hence, concomitant NAFLD not only affects the management of patients with primary liver cancer but also that of patients undergoing resection of liver metastasis from other cancer types (i.e. colorectal cancer).^73^

## HCC in NAFLD and immunotherapy

Immunotherapy with immune checkpoint blockers has been recently added to the treatment armamentarium of HCC.^74^^,^^75^ While monotherapy with programmed cell death protein 1 (PD-1)-targeted antibodies failed in phase III trials in both first-line and second-line,^76^^,^^77^ the combination of atezolizumab plus bevacizumab improved both primary endpoints overall and progression-free survival over sorafenib.^78^ Even though this combination represents the new reference standard in front-line HCC treatment, some patients should still receive tyrosine kinase inhibitors in first-line due to safety reasons (i.e. patients with a history of organ transplantation or severe autoimmune disease).^75^

There is also emerging preclinical and clinical evidence that immunotherapy may be less effective in patients with underlying NAFLD/NASH.^75^ Subgroup analyses from both first-line phase III trials of advanced stage HCC testing nivolumab monotherapy or combined atezolizumab/bevacizumab demonstrated that immunotherapy was more efficacious versus sorafenib in patients with underlying viral etiologies compared with non-viral diseases (including NAFLD).^76^^,^^77^ Similar data were reported in the phase III trial testing pembrolizumab monotherapy versus placebo in sorafenib-pretreated patients with HCC.^76^ A meta-analysis including these three phase III studies with a total of 1656 subjects confirmed that immunotherapy was superior versus control arm in patients with hepatitis-B- and hepatitis-C-related HCC, but not in patients with non-viral underlying etiologies {hazard ratio (HR) [95% confidence interval (CI)] for pooled hepatitis B virus/hepatitis C virus and non-viral: 0.64 (0.48-0.94) and 0.92 (0.77-1.11); *P* of interaction = 0.03}.^79^ Notably, this meta-analysis was not based on individual patient data. In two retrospective cohorts of patients with advanced stage HCC treated with PD-(L)1-targeted immunotherapy (*n* = 130 and *n* = 118, respectively), those with NAFLD/NASH-related HCC had a significantly shorter survival than patients with any other etiology.^79^

**Figure 2:**
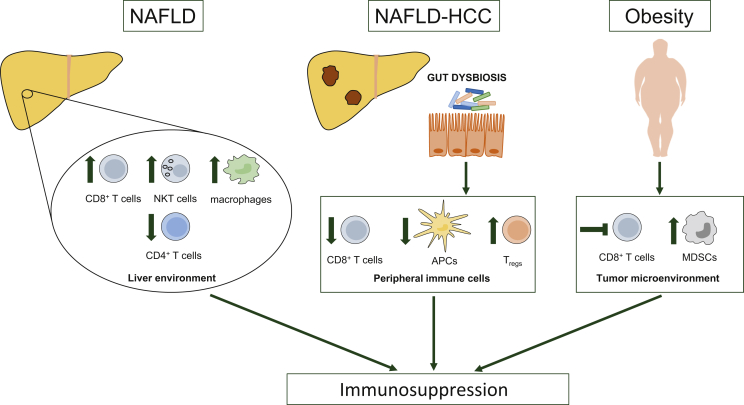
Factors promoting immunosuppression in patients with non-alcoholic fatty liver disease. NAFLD impacts the liver immune microenvironment. While the number of CD4^+^ T cells with antitumor functions is reduced, CD8^+^ T cells, NKT cells, and macrophages with tumor-promoting properties expand in NAFLD. Gut dysbiosis in NAFLD-related hepatocellular carcinoma promotes peripheral immunosuppression, characterized by reduced numbers of CD8^+^ T cells and antigen-presenting cells and expansion of regulatory T cells. Obesity is a risk factor for NAFLD and thus frequently present in patients with NAFLD. Obesity impairs the function of CD8^+^ T cells and enhances the immunosuppressive potency of tumor-infiltrating MDSCs. APCs, antigen-presenting cells; HCC, hepatocellular carcinoma; MDSCs, myeloid-derived suppressor cells; NAFLD, non-alcoholic fatty liver disease; NKT cells, natural killer T cells; T_regs_, regulatory T cells.

Mechanistically, NASH impacts the hepatic immune environment (Figure 2).^80^ For instance, NASH promotes a pro-tumorigenic milieu driven by exhausted, unconventionally activated CD8^+^PD-1^+^ T cells. In mouse models of NASH, anti-PD-1 treatment increased hepatic and tumoral CD8^+^PD-1^+^ T-cell accumulation, but failed to induce regression of liver tumors. Instead, mice experienced enhanced liver damage and hepatocarcinogenesis. Depletion of CD8^+^ T cells decreased anti-PD-1-induced tissue damage and HCC incidence.^79^ Moreover, NAFLD induces loss of hepatic CD4^+^ T lymphocytes, which hampers tumor immunosurveillance and fosters HCC development.^81^ In preclinical models with diet-induced steatohepatitis and intrahepatic injection of melanoma or colon cancer cells, immunotherapy with an RNA-based vaccine or an antibody against OX40 failed to inhibit intrahepatic tumor growth. Tumors of mice with steatohepatitis showed fewer CD4^+^ T cells and effector memory cells compared with tumors of mice on regular diet. Prevention of intratumoral T-cell loss recovered efficacy of immunotherapy.^82^

Gut microbiota has been implicated in modulating response to immunotherapy.^83^ Recent evidence suggests that altered gut microbiome in NASH-related HCC may hamper immunotherapy efficacy by modulating peripheral immune responses. Accordingly, gut dysbiosis in patients with NASH-HCC resulted in peripheral immunosuppression (reduced CD8+ T cells and antigen-presenting cells, increased regulatory T cells)—at least partly via increased short-chain fatty acid production^84^ (Figure 2). Early findings in melanoma patients suggest that approaches to modulate the gut microbiome (i.e. fecal microbiota transplant) may help to overcome immunotherapy resistance and render tumors more susceptible to immune checkpoint blockers (ICBs).^85^^,^^86^

Obesity—typically associated with NAFLD—can hamper antitumor immunity (Figure 2). In murine models, high fat diet (HFD) increased the accumulation of myeloid-derived suppressor cells (MDSCs) via leptin and enhanced the immunosuppressive activity of tumor-infiltrating MDSC; MDSCs enhanced cancer progression by preventing T-cell activation.^87^ Moreover, HFD-induced obesity impaired the function of CD8^+^ T cells via induction of metabolic changes in the tumor microenvironment, resulting in enhanced cancer growth.^88^ In another preclinical study, obesity promoted PD-1-mediated T-cell dysfunction—partly via leptin signaling—and tumor growth. However, obesity was associated with increased responsiveness of tumors to anti-PD-(L)1 treatment,^89^ suggesting that obesity-mediated immunosuppression can be reversed by ICBs. This is in line with several clinical reports showing better response rates and survival for obese patients with advanced cancers treated with immunotherapy.^90^, ^91^, ^92^

Together, these data support the notion that NAFLD is associated with reduced immunotherapy efficacy, not only in HCC but also in hepatic metastases from other tumor entities. Potential deleterious effects of PD-1-targeted therapy on NAFLD progression could also affect immunotherapy-treated patients with extrahepatic cancer types who suffer from concomitant NAFLD. Besides NASH-associated changes of the hepatic immune milieu, gut dysbiosis-related immunosuppression may hamper immunotherapy efficacy in NASH-HCC. If modulation of the gut microbiota can render tumors more susceptible to ICBs needs to be addressed in future studies. Obesity-induced immunosuppression may be reversed by ICBs.

## Chemotherapy-associated steatohepatitis: mechanisms and clinical implications

Chemotherapy-induced acute steatohepatitis (CASH) describes inflammation with hepatocyte injury and steatosis of the liver in patients receiving systemic chemotherapy, possibly leading to liver-related complications such as sinusoidal obstruction syndrome (SOS) or nodular regenerative hyperplasia (NRH).^10^^,^^11^^,^^93^^,^^94^ Most data on chemotherapy-associated steatohepatitis and liver injury comes from patients with colorectal liver metastases where irinotecan- and oxaliplatin-based treatments have been associated with liver injury.^11^^,^^93^^,^^95^ In this setting, liver injury increases post-operative morbidity and liver-surgery-specific complications.^11^ Mainly oxaliplatin treatment was linked to severe sinusoidal dilatation, which resulted in an increased rate of major morbidity.^11^ However, various other systemic chemotherapeutics can induce CASH-like liver injury, the most common being methotrexate, 5-fluorouracil, irinotecan, tamoxifen, and l-asparaginase.^10^ CASH is usually reversible once treatment is stopped. However, liver injury per se can persist for a long time even after cessation of chemotherapy. For instance, while SOS and NRH regressed after 9 months, steatosis and steatohepatitis persisted.^94^ Mechanisms behind CASH are not entirely clear but seem to be based on mitochondrial dysfunction. Mitochondrial and peroxisomal beta-oxidation lead to lipid peroxidation via reactive oxygen species, which induces stellate cell activation, fibrosis, cell death, and ultimately CASH.^10^

Apart from the chosen chemotherapy regimen, obvious risk factors for CASH—which overlap with risk factors for NAFLD—include components of the metabolic syndrome, above-average alcohol intake, and previous chronic liver disease of any etiology.^10^ Additionally, genetic polymorphisms that play a key role in hepatic fat metabolism (i.e. PNPLA3) seem to influence the risk for developing CASH.^96^

CASH can be particularly problematic in patients who underwent downstaging with chemotherapy before resection, as steatohepatitis increases the risk of post-operative morbidity and mortality.^97^^,^^98^ Thus, a risk-benefit assessment regarding tumor progression during the chemotherapy-free period versus the risk for post-operative complications should be done before deciding on the proper timing of surgery.^10^ Generally speaking, the longer the interval between chemotherapy and hepatic resection, the lower the risk of liver-related post-operative complications.^10^^,^^99^

In patients scheduled for a chemotherapy regimen with increased risk of CASH, preexisting liver diseases, potential risk factors for CASH, and pre-treatment liver function should be evaluated. The latter includes blood tests and imaging (i.e. ultrasound), and non-invasive fibrosis assessment (i.e. FIB-4, VCTE) if underlying liver disease is suspected. During chemotherapy, liver function should be monitored on a regular basis. In case of suspected liver injury, other potential causes (i.e. hepatotoxic co-medication, viral hepatitis, autoimmunological liver diseases, alcohol abuse, biliary obstruction, tumor progression) should be excluded. Liver biopsy may be indicated based on the results of non-invasive tests and severity of liver damage. In case of liver surgery after chemotherapy, the condition of the liver should also be evaluated preoperatively (Table 2).^10^

**Table 2:** Chemotherapy-associated acute steatohepatitis (CASH)—bullet points

Chemotherapeutics associated with CASH?	• Methotrexate, 5-fluoruracil, irinotecan, tamoxifen, L-asparaginase
Risk factors for developing CASH?	• Chronic liver disease, metabolic syndrome (obesity, diabetes mellitus, hyperlipidemia, arterial hypertension), genetic risk factors (polymorphism in PNPLA3 genotype)
Diagnostic work-up?	• Medical history • Standard laboratory analysis • Rule-in/-out previously undiagnosed chronic liver disease (CLD) if either (i) medical history or (ii) laboratory markers are indicative for chronic liver disease • If suspicious of CLD → perform diagnostic work-up including abdominal ultrasound, exclusion of other causes of CLD, non-invasive fibrosis scores • Discuss liver biopsy with your local hepatologist: risk/benefit • Discuss risk/benefits of chemotherapy with the patient • DO NOT delay initiation of chemotherapy longer than necessary
Monitoring during chemotherapy?	• Tight monitoring recommended in patients at high risk for CASH during chemotherapy

PNPLA3, Patatin-like phospholipase domain-containing protein 3.

## Conclusion

Hepatic steatosis induced by cytotoxic chemotherapy (CASH) is usually reversible after cessation of therapy, even though it may persist in some cases.^94^ In contrast, NAFLD is a highly prevalent and further increasing liver disease that can progress to liver cirrhosis and HCC. Due to a lack of effective drug treatments, management of NAFLD mainly focuses on lifestyle interventions.^14^ NASH-related HCC often occurs in non-cirrhotic patients with well-preserved liver function, which would be optimal conditions for surgical resection. However, due to the lack of robust screening recommendations in non-cirrhotic NASH patients, tumors are often diagnosed at an advanced stage, where only systemic therapies can be applied.^47^ The recent approval of the combination of atezolizumab plus bevacizumab represents a milestone in the systemic management of patients with advanced stage HCC. Emerging data suggest that changes in the local immune microenvironment and gut dysbiosis may hamper the efficacy of immunotherapy in NASH-HCC.^79^^,^^82^^,^^84^ These data are preliminary and need validation in prospective studies. Hence, based on the current evidence, immunotherapy should not be withheld from patients with NASH-HCC.

Pharmacological therapy for NASH is researched extensively. While we are still waiting for a striking breakthrough, we can only speculate about potential benefits of drugs to reverse hepatic steatosis, inflammation, and fibrosis. In NAFLD patients, they may prevent disease progression to cirrhosis and HCC. They may reprogram the immune microenvironment in patients with NASH-HCC, which could have implications on treatment efficacy and outcome. Depending on the mode of action (e.g. anti-inflammatory), some of these drugs could even be tested as a prophylactic treatment in cancer patients with a high risk for CASH.
